# Immune landscape and in vivo immunogenicity of NY-ESO-1 tumor antigen in advanced neuroblastoma patients

**DOI:** 10.1186/s12885-018-4910-8

**Published:** 2018-10-16

**Authors:** Chiara Camisaschi, Salvatore Lorenzo Renne, Valeria Beretta, Francesca Rini, Rosalin Dolores Spagnuolo, Alessandra Tuccitto, Marta Giorgia Podda, Giorgio Parmiani, Licia Rivoltini, Paola Collini, Chiara Castelli, Roberto Luksch

**Affiliations:** 1 Department of Experimental Oncology and Molecular Medicine, Unit of Immunotherapy of Human Tumors, Fondazione IRCCS Istituto Nazionale dei Tumori, Milan, Italy; 2 Department of Diagnostic Pathology and Laboratory Medicine, Soft Tissue and Bone Pathology, Histopathology and Pediatric Pathology Unit, Fondazione IRCCS Istituto Nazionale dei Tumori, Milan, Italy; 3 Department of Pediatric Oncology, Fondazione IRCCS, Istituto Nazionale dei Tumori, Via G. Venezian 1, 20133 Milan, Italy

**Keywords:** Neuroblastoma, NY-ESO-1, Vaccination, Immune-checkpoints, Immune-contexture

## Abstract

**Background:**

Indirect evidence suggesting the immunosensitivity/immunogenicity of neuroblastoma is accumulating. The aims of this study were to investigate the immune landscape of neuroblastoma and to evaluate the in vivo immunogenicity of the NY-ESO-1 tumor antigen in advanced neuroblastoma patients.

**Methods:**

The immune infiltrating cells of the NY-ESO-1+ tumors from three HLA*A201 patients with metastatic neuroblastoma who relapsed after conventional treatments were evaluated by immunohistochemistry. The patients were vaccinated with the HLA-A*0201-restricted peptide NY-ESO-1_157-165(V)_. The peptide was emulsified in Montanide ISA51 and given subcutaneously in a phase I pilot study. The immunogenicity of NY-ESO-1 antigen was evaluated by monitoring mononuclear cells in patient peripheral blood, pre- and post-vaccine, by short-term in vitro sensitization, HLA-multimer staining and IFN-γ ELISpot analysis.

**Results:**

Both CD3 T cells and CD163 myeloid cells were present in pre-vaccine tumors and PD-1 and PD-L1 expression was mainly found in the immune infiltrate. Despite the advanced stage of the disease, the vaccination induced systemic NY-ESO-1 specific CD8 T cells releasing IFN-γ in response to activation with the NY-ESO-1 peptide and an HLA-A2 positive neuroblastoma cell line.

**Conclusions:**

Our results indicate that vaccination with a tumor-associated peptide is able to boost NY-ESO-1-specific, functionally active T cells in advanced neuroblastoma patients with lymphocyte infiltration in their pre-vaccine tumors.

**Trial registration:**

EudraCT #2006–002859-33.

**Electronic supplementary material:**

The online version of this article (10.1186/s12885-018-4910-8) contains supplementary material, which is available to authorized users.

## Background

Metastatic neuroblastoma (NBL) diagnosed in patients over 18 months of age has a poor prognosis and is classified as “high-risk NBL”. Intensive induction regimens, the use of myeloablative treatment and autologous hematopoietic stem cell rescue do not prevent a high incidence of relapse [1]. The chance of cure for high-risk NBL patients is extremely low, and a standard therapy does not exist [2]. Thus, novel therapeutic approaches are urgently needed.

There is increasing evidence suggesting the immunosensitivity/immunogenicity of NBL. It has been recently reported that tumor-infiltrating T cells have prognostic value in NBL, and that combined PD-L1 and HLA-class I expression on tumor cells predicts the clinical outcome in NBL patients [3, 4]. Moreover, NBL cells express tumor-associated antigens (TAA), such as MAGE and NY-ESO-1, which are specific targets for humoral and T cell mediated response [5–7].

Based on this experimental evidence, several ongoing clinical studies are investigating different immunological interventions such as cytokine/chemokine treatments, antibody administration, cancer vaccines and adoptive therapy with lymphocytes genetically engineered to acquire NBL specificity [8]. GD2, a surface glycolipid, is the most common target for antibody-based immunotherapy in NBL [9]. Among immunotherapy approaches, vaccines have made a comeback and their use in combination with immune-checkpoint inhibitors (ICI) is under evaluation for a variety of solid tumors [10–12]. Different types of vaccination strategies, including vaccines with genetically modified NBL cells, DC or DNA vaccines, have been applied to preclinical models of NBL, and some of them have been translated into clinical experimentation. Preclinical studies indicate that vaccines may control the growth of NBL tumor in animal models [13–16]. Moreover, local or systemic anti-tumor T cell responses have been often observed in NBL vaccinated patients [17–19]. New clinical trials with DC loaded with NBL antigens (cell lysates, tumor RNA, TAA) are either ongoing (NCT02745756; NCT00405327) or have recently been completed (NCT01241162).

Interestingly, encouraging results were obtained with vaccination aimed at inducing a specific antibody-mediated immune response against GD2 and GD3 in NBL patients experiencing 2nd or further complete/very good partial remission. The majority of these patients developed specific anti-GD2 and -GD3 antibody response [20]. A larger phase II study testing the clinical efficacy of this immunotherapy is currently ongoing (NCT00911560).

A pilot phase I trial of tumor lysate-pulsed DCs conducted in pediatric patients with solid tumors showed that the frequency of anti-tumor T cells was greatly increased in the peripheral blood of a subset of vaccinated patients, including NBL patients [21, 22]. Moreover, the induction of this tumor-specific immunity was associated with a condition of stable disease that lasted for 16–30 months.

All these results hold promise for the application of vaccine therapy in NBL patients, nevertheless suitable antigenic targets, optimal adjuvant formulations and administration route have not been defined yet.

In terms of possible TAA for NBL, we reported that NY-ESO-1, a germ cell antigen aberrantly expressed in different tumor types, is present in NBL tumors at diagnosis. We documented that a proportion of NBL patients show natural, humoral and T-cell mediated response against NY-ESO-1 protein and the HLA-A2-restricted NY-ESO-1_157-167_ peptide [23]. Moreover, T cells engineered to express NY-ESO-1 specific TCR not only kill NBL cells in vitro but also limit the in vivo tumor progression of localized and disseminated NBL xenografts [24]. All these findings support the relevance of NY-ESO-1 protein as a candidate antigen for vaccine development.

Here we describe the immune landscape of the primary tumor of three high-risk NBL patients who received the NY-ESO-1_157-167(V)_ synthetic peptide vaccine in a pilot single-center study protocol. Our study shows that vaccine can boost NY-ESO-1 specific immunity in patients whose tumor microenvironment is infiltrated by lymphocytes.

## Methods

### Patients

A pilot study of peptide vaccination in HLA-A*0201 NBL patients was carried out at Fondazione IRCCS Istituto Nazionale dei Tumori as a monoinstitutional study protocol (EudraCT #2006–002859-33). The study was conducted in compliance with the Declaration of Helsinki and approved by the Institutional Review Board. The parents provided informed consent on behalf of the patients in all cases.

The criteria of eligibility included: diagnosis of histologically proven NBL, > 1 year of age at diagnosis, stage 4 relapsed tumor or resistant disease after conventional therapies, tumor positivity for NY-ESO-1 expression assessed by immunohistochemistry (IHC), HLA typed as HLA-A*0201, absence of concurrent immunosupressive treatments (e.g. steroids, cyclosporine), absence of any concurrent serious or immunosuppressive medical condition (e.g. AIDS, autoimmune diseases) and absence of concurrent second malignant tumor.

The evaluation of the extent of disease at study entry was performed with whole-body CT scan, 123-I-mIBG scintigraphy, bilateral bone marrow aspirate and biopsy, and urinary catecholamine levels.

Patients were checked for HLA-A*0201 expression using the Olerup SSP HLA Kit (Qiagen S.p.A).

Among a cohort of 28 consecutive NBL patients identified as potential candidates, 11 were positive for NY-ESO-1 expression. NY-ESO-1 expression was defined as positive if at least 1% of the tumor cells had intensity ≥1 on a 0–3 scale. The intensity of positive staining was scored as 0 if not visible at any amplification, as 1 if neatly visible at 20-40X, 2 if neatly visible at 10X and 3 if neatly visible at 4X (ocular 10X). The ‘NY-ESO-1 score’ for the 11 NBL NY-ESO-1 positive tumors, calculated as the percentage of positive cells x intensity score, is reported in (Additional file 1: Table S1). (Additional file 2: Figure S1 (A)) shows representative images of NY-ESO-1 expression in the cutaneous primary melanoma Me5810 used as positive control, and in NBL tumors scored as 1 and 2. NY-ESO-1 expression in NBL tumors was independent of the degree of tumor differentiation; positivity of the NY-ESO-1 antigen was detected both in differentiating and undifferentiating NBL cells (Additional file 2: Figure S1(B)). Five of the patients with NY-ESO-1+ tumors were typed as HLA-A*0201.

The trial was closed shortly after its start because the Manufacturer (Clinalfa Merck Bioscience AC) changed ownership and could no longer provide any GMP certificated peptide batches. Thus, only three out of five patients typed as HLA-A*0201 were enrolled in the study and received the vaccine.

### Vaccine preparation and vaccination protocol

The vaccine formulation included the altered peptide ligand (APL) NY-ESO-1_157-165(V)_ (260 μg, Merck Biosciences) emulsified in Montanide ISA51 (0.25 mL, Seppic), and diluted in physiological solution (0.25 mL, Diaco), and was administered by subcutaneous injection. The peptide (> 95% pure) was synthesized under GMP conditions by Merck Biosciences AG Clinalfa.

**Fig. 1:**
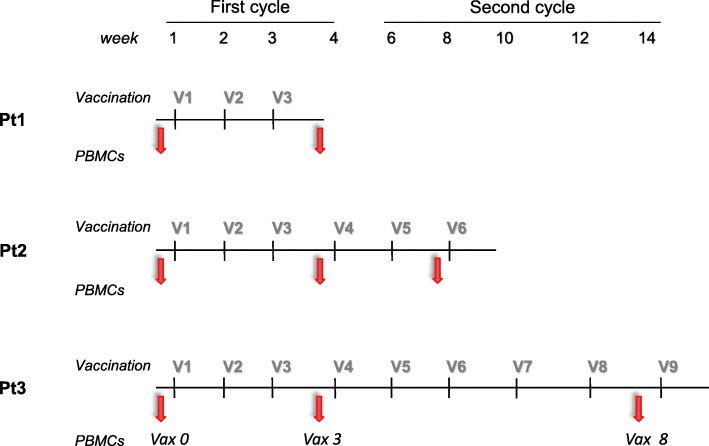
Vaccination schedule and PBMC collection. The vaccination consisted of two cycles: the first one at weekly administration × 4 weeks (First cycle) and the second with fortnightly administration × 5 (Second cycle), for a total of nine administrations. Pt = Patient; V = vaccine administration; PBMCs = peripheral blood mononuclear cells; Arrows = PBMC collection

The treatment schedule is shown in Fig. 1. It consisted of two cycles of vaccination administered subcutaneously. In the first cycle, vaccine was given weekly for four times, and in the second cycle fortnightly for five times, for a total of nine administrations or until disease progression. The treatment schedule and doses were established based on previous experiences demonstrating the successful induction of persistent peptide-specific T cell responses in adult cancer patients [25, 26].

During the entire vaccination period, physical evaluation plus complete blood chemistry and urinary catecholamines were evaluated twice a week during the first cycle and every two weeks thereafter.

A complete re-evaluation according to INRC criteria [27] was programmed after the 9th administration of the vaccine, or when disease progression occurred. Follow-up re-evaluations were scheduled every 2 months for 3 times and thereafter every 6 months for 5 years. In case of a doubling of HVA/VMA levels without any radiological evidence of relapse, clinical follow-up was planned at monthly intervals.

The toxicity of the vaccination was recorded according to the National Cancer Institute’s Common Terminology Criteria for Adverse Events (NCI-CTCAE v3.0, June 2003; http://ctep.cancer.gov/forms/CTCAEv3.pdf; Appendix II). Any grade 3–4 toxicity, with the exception of symptomatic macular, papular or vesicular skin eruption at the site of vaccine injection (grade 3 skin toxicity), or any grade 1–2 toxicity requiring medicalization that could interfere with the immune response (e.g. steroids) were considered as severe adverse events.

### Immunomonitoring

Blood samples (15 mL) were obtained from each patient at the time points indicated in Fig. 1, always before the vaccine administration. PBMCs were isolated within 3 h after blood draw using Ficoll-Paque™ PLUS density gradient centrifugation as previously described [28]. The cells were stored in the vapor phase of a liquid nitrogen vessel until further use. In vitro short-term peptide sensitization (10 to 14 days) was induced by stimulating the thawed PBMCs with the vaccine APL NY-ESO-1_157-165(V)_ (3 μg/ml) or with HIV NEF_180–189_ peptide (3 μg/ml, Immatics), in the presence of 60 IU/mL of IL-2 (Proleukin).

IFN-γ-ELISpot assay (1-D1K, Mabtech AB) was performed on peptide-sensitized PBMCs in the presence of T2 cells pulsed with the APL NY-ESO-1_157-165(V)_ or with the irrelevant HIV NEF_180–189_ peptide (1 μg/ml) or in the presence of NBL cell line ACN expressing the HLA-A*0201 (ACN-A2) (kindly provided by Dr. Vito Pistoia), as previously described [26]. HLA blocking experiments were carried out by preincubating the ACN-A2 target cells with the anti–class I HLA (A, B, and C) immunoglobulin M antibody (clone A6–136; kindly provided by Dr. Daniela Pende, INT, Genoa, Italy). Briefly, patient PBMCs were washed, resuspended in complete medium (RPMI 10% FCS) and seeded in 3 replicate wells at a density of 1X10^5^ cells per well in a Multiscreen 96-well plate (MAIPSWU10, Millipore) coated with the anti-IFN-γ antibody (1-D1K, Mabtech). Antibody incubations and the development of the ELISpot assay were according to the manufacturer’s instructions (Mabtech). The spots were counted with an ELISpot Reader Instrument (Aelvis-Tema). Results are presented as the number of APL-reactive cells / 2X10^5^ cells.

Pro5 HLA-A*0201/NY-ESO-1 multimer (SLLMVVITQV) and the irrelevant HLA-A*0201/HIV multimer (HIV- Gag p24; TLNAWVKVV) were provided by Proimmune Ltd. Fluorochrome-conjugated anti-CD8 and anti-CD19 monoclonal antibodies were from BD Biosciences. The staining was performed according to the manufacturer’s instructions. Cell samples were acquired on a BD FACSCalibur (BD Biosciences) or Gallios flow cytometer (Beckman Coulter) and analyzed using FlowJo software (TreeStar Inc.).

### Immunohistochemistry

Formalin-fixed, paraffin-embedded (FFPE) tumor tissues obtained from each patient before the vaccination were analyzed. Serial 2-μm thick sections were cut and processed for IHC. Primary antibodies used: CD3-Policlonal (A045201–2, 1:400), CD8 (C8/144B, 1:20), CD68-KP1 (M081401–2, 1:3000) and CD68-PGM-1 (M087601–2, 1:50), all purchased from Dako; CD163-10D6 (NCL-CD163, 1:200), GZMB (11F1, 1:80) (Novocastra, Leica Biosystems); Tbet (4B10, 1:80) (Santa Cruz); MHC-I-EMR8–5 (Ab70328, 1:4000) (Abcam); NY-ESO-1-E978 (35–6200, 1:200) (Thermofisher); PD1-NAT105 (3137, 1:50) (Biocare) and PDL1-RBT (BSB2654, 1:50) (Bio SB). All these primary antibodies were processed using the Autostainer Link 48 Dako System.

Immune infiltrating cells were quantified by counting the number of immune reactive cells at 400X magnification. The three areas with the most intratumor inflammatory cells were selected. Results are reported as mean value of immune reactive cells/mm^2^.

### Statistical considerations

For ELISpot assays, values were compared using unpaired Student’s t-test (significant *p* value ≤0.05). Statistical calculations were carried out using Prism5 (GraphPad Software).

## Results

### Patient description

All patients received the NBL-HR1 Study Protocol of SIOP-Europe neuroblastoma as first-line treatment [29] and entered the vaccine protocol at least 1 month following therapy completion.

Patient #1 was a girl with MYCN amplified NBL. She was 5 years old and entered the study at the first relapse. She received three vaccinations of the first cycle. She was subsequently withdrawn because she experienced a rapid progression of the disease, with fatal outcome 4 months after the study entry.

Patient #2 was a girl, with MYCN non-amplified tumor. She entered the study when she was 3 and a half years old, with skeletal and bone marrow relapse. She completed the first cycle of vaccinations and received the first two vaccinations of the second cycle. She was withdrawn after progression of disease in the bone marrow, and she died of disease 2 months later.

Patient #3 was a girl with MYCN non-amplified tumor. The patient entered the study at 5 years of age with progressive disease after topotecan + vincristine + doxorubicin treatment. She completed both vaccination cycles and remained progression-free throughout the 14 weeks of vaccine administration and for the following 4 weeks. At week 18, disease progression occurred leading to death 5 months later.

The vaccination was well tolerated by all the patients; no adverse events were recorded. In Patient #2 and Patient #3, the only documented side effect was an expected transient local reaction at the injection site, consisting of itching and grade 1 erythema according to CTCAE v.3.0. This local reaction appeared a few minutes after all the inocula of the second cycle; it was transient and did not require any additional treatment.

### Presence of immune infiltrating cells in the tumor

**Fig. 2:**
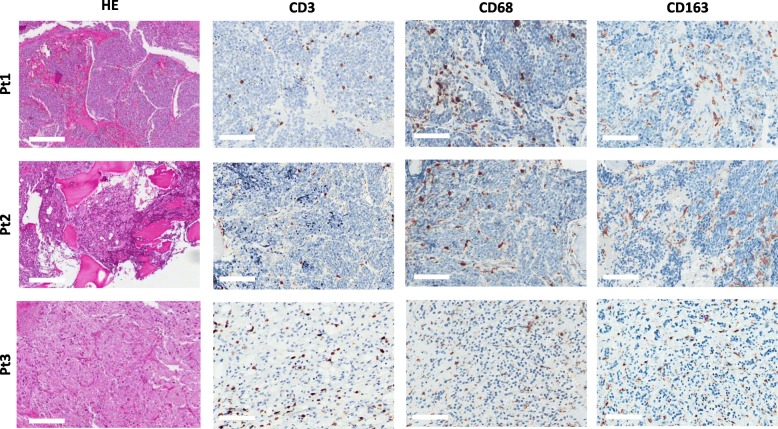
Presence of immune infiltrating cells in NBL before the vaccination. IHC was performed on consecutive sections of FFPE tumor samples. Representative hematoxylin-eosin (HE) and IHC images of intratumoral CD3, CD68 and CD163 positive cells are shown for the three enrolled patients: Pt#1, 2, 3. Scale bar = 200 μm for HE and 100 μm for the other panels. For higher magnification see Additional file 3: Figure S2

The immune tumor-infiltrating cells from the patients enrolled in the study were investigated by IHC performed on consecutive sections of FFPE samples of tumor obtained after chemotherapy treatments and before the start of the vaccination schedule. In all patients, CD3 lymphocytes were detected and localized within the tumor cell nests and/or in surrounding fibrovascular septa. The tumor areas were also invaded by tumor associated macrophages (TAM), positive for CD68 and CD163 markers (Fig. 2 and Additional file 3: Figure S2). TAM were particularly enriched in a necrotic area of the tumor from Patient #3 displaying sclerohyalinosis, fibrous reaction and hemosiderin accumulation that was likely a result of the chemotherapy treatments (Additional file 4: Figure S3).

**Fig. 3:**
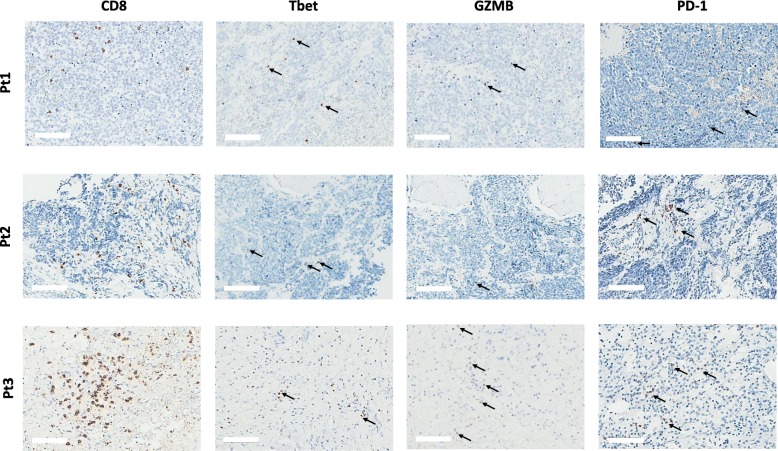
Characterization of CD8 infiltrating cells in NBL before the vaccination. IHC was performed on consecutive sections of FFPE tumor samples that were stained for Tbet, GZMB and PD-1 markers. Examples of immune infiltrating cells with nuclear Tbet, granular cytoplasmic GZMB or membrane PD-1 positive staining are indicated by arrows. Scale bar = 100 μm. For higher magnification, see Additional file 5: Figure S4

IHC also showed that the CD3+ cells infiltrating the pre-vaccine tumors of all patients included CD8+ lymphocytes (Fig. 3). Nuclear expression of Tbet, cytoplasmic decoration for Granzyme B (GZMB) and membrane expression of PD-1 were detectable in immune infiltrating cells of all three tumors, albeit with a low frequency, with Patient #3 showing the highest numbers (Fig. 3 and Additional file 5: Figure S4), Table 1).

**Table 1:** Assessment of intratumoral inflammatory cells expressing immune related markers

Immune markers	Patient 1	Patient 2	Patient 3
CD3	127^a^	106	185
CD8	98	83	170
Tbet	13	< 4	27
GZMB	< 4	< 4	12
CD68	137	80	257
CD163	104	115	153
PDL-1	11	< 4	< 4
PD-1	< 4	6	16

^a^Number/mm^2^ of immune tumor-infiltrating cells expressing the indicated markers. See Materials and Methods for details

**Fig. 4:**
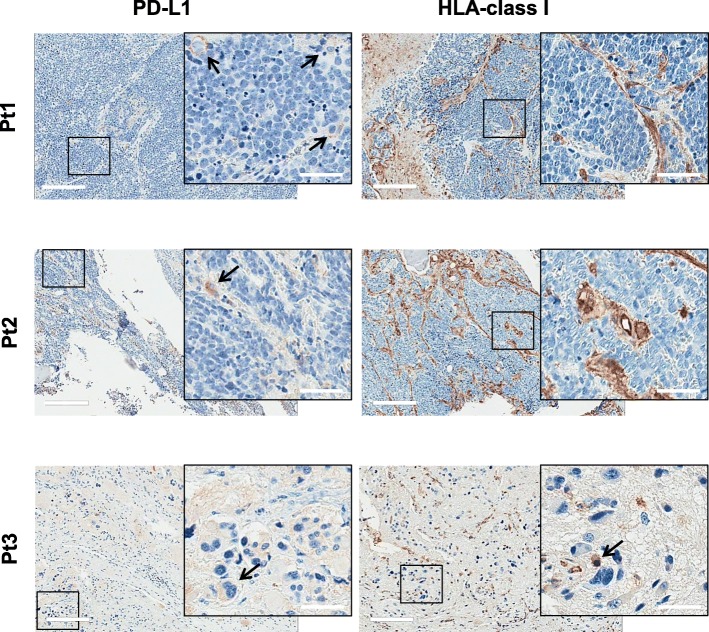
PD-L1 and HLA-class I expression in NBL before the vaccination. IHC was performed on consecutive sections of FFPE tumor samples before the vaccination. Representative IHC images of PD-L1 and HLA-class I markers for the three enrolled patients (Pt1, 2, 3) are reported. Arrows indicate PD-L1 and HLA-class I positive tumor cells. Scale bar = 200 μm; inserted panels scale bar = 50 μm

Since a strong presence of tumor infiltrating T cells together with detectable levels of PD-L1 and tumor neoantigens is predictive of response to anti-PD-1/PD-L1 therapy in cancer patients [30, 31], we analyzed the expression of PD-L1 in the NBL microenvironment. PD-L1 positive neoplastic cells were not found in any of the tumors analyzed. PD-L1 expression was only detected in few tumor-infiltrating inflammatory cells displaying macrophage morphology and characterized by abundant granular cytoplasm, small nucleus and a low nucleus/cytoplasm ratio (Fig. 4). In the three examined NBL tumors, IHC revealed the presence of few HLA-class I positive cancer cells, whereas, as expected, immune-infiltrating cells were HLA-class I positive (Fig. 4).

Table 1 summarizes the IHC characterization of intratumoral immune cells.

### Rapid and persistent induction of functional, peptide-specific CD8 T cells in post-vaccine PBMCs

**Fig. 5:**
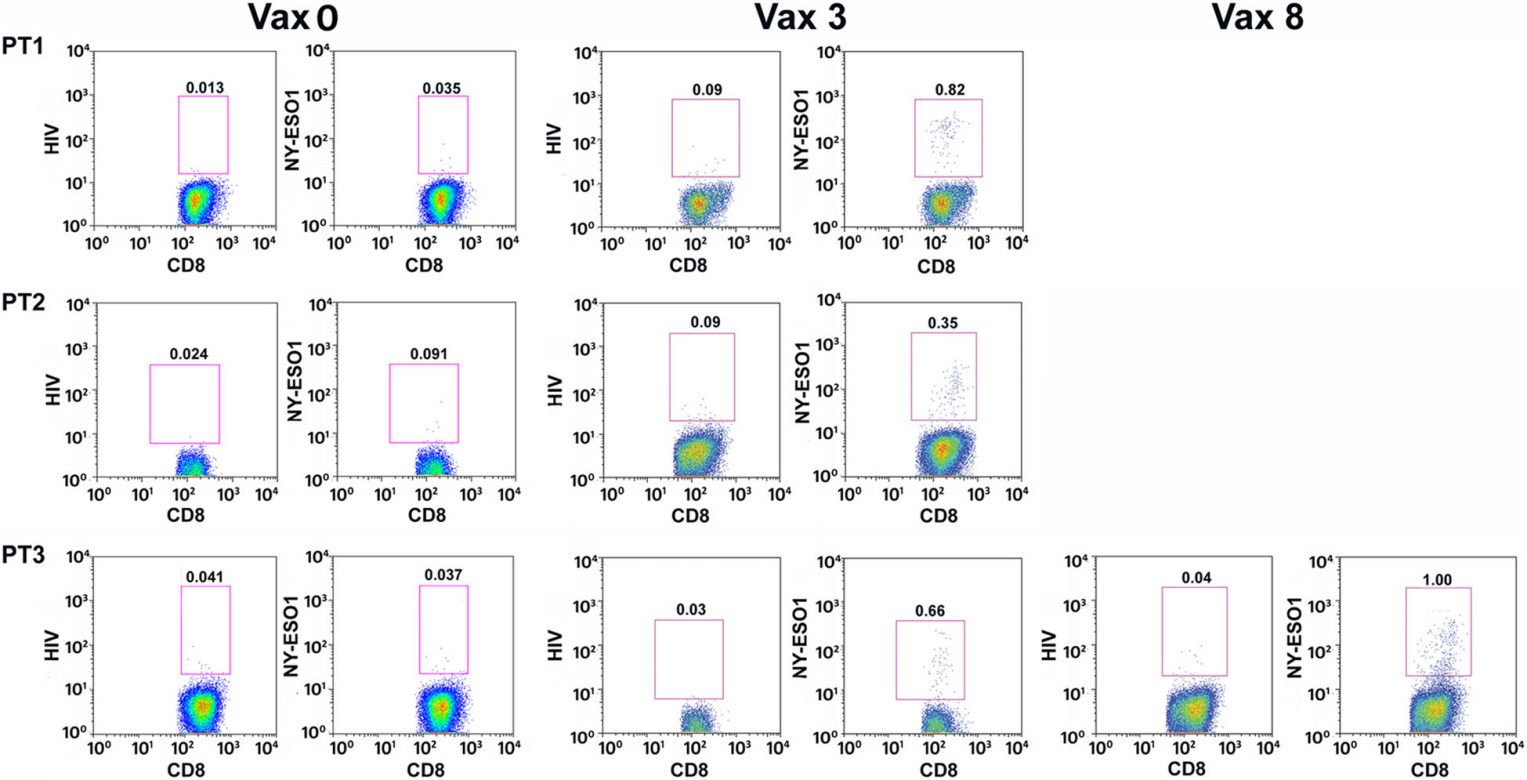
Rapid and persistent induction of antigen-specific CD8+ T cells during the vaccination. The PBMCs of Pt #1, 2 and 3 collected pre- or post-vaccination at different time points were analyzed using HLA-A*0201/NY-ESO-1 or HLA-A*0201/HIV-p24 multimer staining as negative control. The percentage of CD8+ multimer positive cells was calculated in the CD8 + CD19- gate and reported in figure. Vax 0 = pre-vaccination; Vax 3 = post 3rd vaccine administration; Vax 8 = post 8th vaccine administration

To assess whether systemic NY-ES0–1 specific immunity can be boosted in high-risk NBL patients, we measured the number of T cells reactive to NY-ESO-1 peptide in the peripheral blood of patients pre- and post-vaccination. The frequency of NY-ESO-1 multimer-specific CD8 T cells, undetectable in pre-vaccine PBMCs, increased in all patient PBMCs collected after the 3rd vaccination, with a frequency of NY-ESO-1 specific CD8 T cells ranging from 0.35 to 0.82% (Fig. 5). In Patient #3, NY-ESO-1 multimer-specific CD8 T cells were further increased by the subsequent vaccinations and reached the frequency of 1% after the 8th vaccination (Fig. 5).

**Fig. 6:**
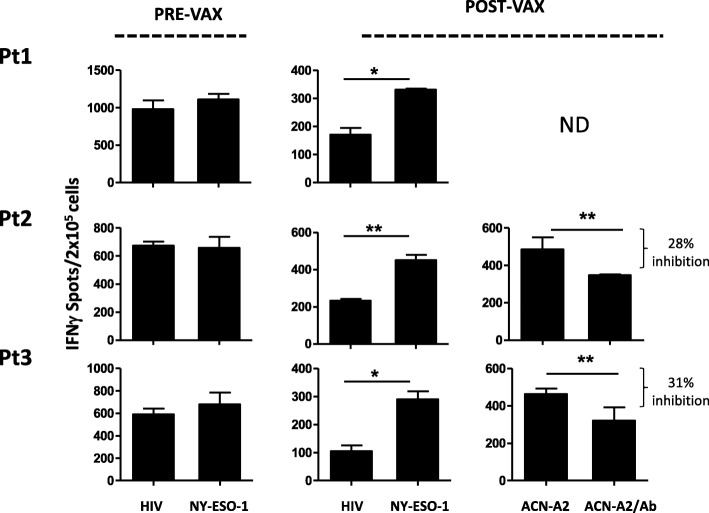
Post-vaccine PBMCs contain cells releasing IFN-γ upon NY-ESO-1 peptide stimulation. Cells releasing IFN-γ in response to HIV or to NY-ESO-1 peptide were detected by Elispot assay in the NBL patient PBMCs collected pre-vaccine and at different time points during vaccination (Pt1 and Pt2, Vax3; Pt3, Vax8). PBMCs post-vaccine were also stimulated with the ACN-A2 cell line, a NBL cell line stably expressing the HLA-A*0201 molecules, in the absence (ACN-A2) or presence (ACN-A2/Ab) of the blocking anti-class I HLA-A, B and C immunoglobulin M antibody (clone A6/136). Results are reported in graph as the number of IFN-γ spots / 2 × 10^5^ cells. The *p* value was calculated using Student’s t-tests. Only the significant *p* values are shown (* ≤ 0.05; ** < 0.01)

PBMCs collected before and after the treatments were also monitored ex-vivo for the functional activity of vaccine-induced T cells using IFN-γ-ELISpot assays. Data showed that post-vaccine PBMCs contained T cells specifically releasing IFN-γ upon NY-ESO-1 peptide stimulation (post the 3rd vaccination for Patient #1 and 2, and post the 8th for Patient #3). Conversely, no NY-ESO-1 specific activity was evidenced in pre-vaccination PBMCs. Thus, the NY-ESO-1 multimer positive CD8 T cells were functionally responsive to stimulation by their nominal antigen. Moreover, the post-vaccine PBMCs of two out of three patients also recognized an HLA-A*0201 matched NBL cell line in a HLA-Class I dependent fashion (Fig. 6), likely suggesting a potential reaction of vaccine-stimulated lymphocytes against patient tumor.

## Discussion

In several human cancers, tumor-infiltrating immune cells have prognostic value [32]. Although NBL has long been considered a poorly immunogenic tumor, recent studies demonstrated that immune contexture can influence the behavior of NBL and might therefore be of clinical importance for patient treatment [33]. In particular, the density and localization of tumor-infiltrating CD3 T cells seem to predict patient clinical outcome [3]. Our IHC characterization of NBL tumors obtained from patients with dismal prognosis demonstrated that both innate and adaptive immune infiltrating cells were present in the tumor microenvironment before the vaccination. Immune infiltration of CD3 T cells and a substantial fraction of infiltrated CD8 T cells, were detected in all cases. A fraction of these lymphocytes was PD-1+ suggesting that they were antigen experienced cells. All the NBL tumors examined were also enriched in CD68 and CD163 TAM. We can therefore conclude that the pre-treated high-risk NBL tumors we studied did not belong to the class of immune-desert or immune-excluded tumors as defined by Chen and colleagues [34]. Our IHC data delineated an immune landscape with features of inflamed tumor, thereby providing the rationale to treat NBL patients with immune-based therapy.

All three NBL tumors showed a significant CD8+ infiltration, and a proportion of infiltrating lymphocytes were positive for Tbet and GZMB suggesting a local, potentially activated T-mediated immunity endowed with effector functions. Nevertheless, our IHC data also pointed to the possibility of an immunosuppressive environment. Although PD-L1 was rarely expressed by tumor cells, it was nonetheless detectable in a few cells with macrophage morphology possibly creating an immunosuppressive environment and fostering cancer progression [35, 36].

PD-L1 and HLA-class I expression levels in NBL tumor environment predict patient outcome [4], likely suggesting a role for cell-cell interactions governed by IC. It has been recently reported that Sipuleucel-T or DNA based vaccine fosters the inflammatory response at the tumor site in prostate cancer patients and enhances the local expression of PD-1 and PD-L1, which suggests a rationale for combination therapy with ICI [37–39]. In this study, we were unable to perform analysis of post-vaccine tumors because of ethical reasons. Therefore, we have no indications about the ability of NY-ESO-1 based vaccine to boost PD-1/PD-L1 expression in NBL tumors. Little is known about the therapeutic potential of ICI in NBL, and the present study does not clarify how the efficacy of the vaccination would be improved by combined ICI therapy, but our data warrant further investigations in the field, also considering that IC are emerging as potential effective targets in pediatric tumors as well [40, 41].

In this study, we showed that vaccination with a TAA peptide, the NY-ESO-1_157-167(V)_, succeeded in inducing an antigen specific response in all three studied patients. Even if the number of patients enrolled was limited, our data clearly indicate that, despite the advanced disease stage, the immune system of high-risk NBL patients could be functionally active and boosted to react against the tumor.

Interestingly, Patient #3 developed a systemic NY-ESO-1 specific immunity that lasted for the whole vaccination course. Her tumor, obtained post-chemotherapy and pre-vaccination, showed the highest frequency of tumor infiltrating immune cells and the presence of a tumor area with signs of pathological response to chemotherapy. It is known that chemotherapy induces immunological cell death [42] and promotes both the expression of cancer germline genes, including NY-ESO-1, and the up-regulation of HLA-class I/II as well as co-stimulatory molecules on tumor cells [43]. Thus, we may speculate that, in this Patient, chemotherapy favored a natural anti-tumor immune response subsequently amplified by the vaccination.

Although our data indicate that an antigen-specific response could be elicited also in high-risk NBL patients, the best setting to achieve clinical benefits for immune-based treatments would actually be in patients with lower disease stages and burden of disease. This conclusion also arises from a recent phase I clinical trial, in which pediatric patients with relapsed or therapy-refractory NBL and sarcoma were treated with decitabine and a DC vaccine targeting MAGE-A1, MAGE-A3 and NY-ESO-1 [44]. In Krishnadas study (2015), objective responses, disease stabilizations and the longest progression-free survival occurred in patients with minimal disease burden at the study entry. Additional supporting evidence stems from the administration of a GD2- and GD3- based vaccine to NBL patients in complete/very good partial remission [20]. Results of the study by Kushner et al. clearly indicated that the vast majority of the patients developed vaccine-induced antigen specific humoral immunity. Importantly, minimal residual disease (MRD) responses were detected in more than half the patients, further supporting the notion that immune therapies are likely to be most effective in the MRD status.

## Conclusion

Our data highlight the importance of exploring the therapeutic role of immune-based interventions in patients with NBL and provide the rationale for vaccinating high-risk NBL patients with the NY-ESO1_157–165(V)_ peptide, after or in combination with the chemotherapy treatments usually administered to treat these patients in the absence of a standard therapy.

Drawing conclusions on the vaccine clinical efficacy was not an endpoint of our study because of the limited number of patients enrolled. This notwithstanding, our data suggest that NY-ESO-1 peptide vaccination might represent a possible strategy for high-risk NBL patients.

## Additional files

Additional file 1: **Table S1.** NY-ESO-1 score of NBL tumors expressing NY-ESO-1. In the Table, the ‘NY-ESO-1 score’ for the 11 NBL NY-ESO-1 positive tumors is reported. (DOCX 14 kb)

Additional file 2: **Figure S1.** NY-ESO-1 expression in NBL. IHC was performed on FFPE tumor samples obtained before the time of study entry. (A) Representative expression of the NY-ESO-1 marker in a primary cutaneous melanoma (Me5810, positive control) and in two NBL tumors scored as 1 and 2 (for details see Methods and Additional file 1 Table 1S). Scale bar = 200 μm, left panels; scale bar = 50 μm, right panels. (B) NY-ESO-1 expression in NBL tumor cells is detectable both in differentiating and undifferentiating NBL tumors (upper and lower panel respectively). Scale bar = 50 μm. (PDF 512 kb)

Additional file 3: **Figure S2.** Presence of immune infiltrating cells in NBL before the vaccination. IHC was performed on consecutive sections of FFPE tumor samples. Representative IHC images of intratumoral CD3, CD68 and CD163 positive cells of the tumors of the three enrolled patients: Pt1, 2, 3. Scale bar = 50 μm. (PDF 527 kb)

Additional file 4: **Figure S3**. NBL tumor of Patient #3 showed area with pathological response to chemotherapy. HE and IHC analysis of the CD68 and CD163 macrophage markers of pre-vaccine, post-chemotherapy FFPE tumor of Patient #3. Images representative for tumor area displaying post-treatment changes with sclerohyalinosis, fibrous reaction with macrophages, and hemosiderin are reported. For HE scale bar = 200 μm, left panel, and 100 μm, right panel; for the IHC of CD68 and CD163, scale bar = 100 μm, left panel, and 50 μm right panel. (PDF 470 kb)

Additional file 5: **Figure S4.** Characterization of CD8 infiltrating cells in NBL before the vaccination. IHC was performed on consecutive sections of FFPE tumor samples that were stained for Tbet, GZMB and PD-1 markers. Examples of immune infiltrating cells with nuclear Tbet, granular cytoplasmic GZMB staining or membrane PD-1 staging of are indicated by the arrows. Scale bar = 50 μm. (PDF 717 kb)

## Abbreviations

ALP: Altered Peptide Ligand
CD: Cluster of differentiation
DC: Dendritic cell
DNA: Deoxyribonucleic acid
FFPE: Formalin-Fixed Paraffin-Embedded
GZMB: Granzyme Beta
HE: Hematoxylin-Eosin
HLA: Human Leukocyte Antigens
IC: Immune-Checkpoints
ICI: Immune-Checkpoint Inhibitors
IFN-*γ*: Interferon gamma
IHC: Immunohistochemistry
MAGE: Melanoma-associated antigen
MDR: Minimal Residual Disease
NBL: Neuroblastoma
NY-ESO-1: New York ESOphageal squamous cell carcinoma 1
PBMCs: Peripheral Blood Mononuclear Cells
PD-1: Programmed cell death-protein 1
PD-L1: Programmed cell death-ligand 1
RNA: Ribonucleic acid
TAA: Tumor-Associated Antigen
TAM: Tumor-Associated Macrophage
