# Supplementary material for: Immune landscape and in vivo immunogenicity of NY-ESO-1 tumor antigen in advanced neuroblastoma patients
Source: BMC Cancer. 2018 Oct 16;18:983. doi: 10.1186/s12885-018-4910-8 (PMC6192300; doi:10.1186/s12885-018-4910-8)
Supplement: Supplementary file 1 — Table S1. NY-ESO-1 score of NBL tumors expressing NY-ESO-1. In the Table, the ‘NY-ESO-1 score’ for the 11 NBL NY-ESO-1 positive tumors is reported. (DOCX 14 kb) [file 12885_2018_4910_MOESM1_ESM.docx]

**Table S1. NY-ESO-1 score of NBL tumors expressing NY-ESO-1.**

| Patient # | HLA-A*0201 typing | % of NY-ESO-1 positive cells | NY-ESO-1 intensity score | NY-ESO-1 score |
| --- | --- | --- | --- | --- |
| 1 | Positive | 100 | 3 | 300 |
| 2 | Positive | 1 | 2 | 2 |
| 3 | Positive | 1 | 2 | 2 |
| 4 | Negative | 10 | 1 | 10 |
| 5 | Negative | 50 | 2 | 100 |
| 6 | Negative | 10 | 1 | 10 |
| 7 | Negative | 20 | 2 | 40 |
| 8 | Negative | 1 | 1 | 1 |
| 9 | Negative | 1 | 1 | 1 |
| 10 | Positive | 10 | 1 | 10 |
| 11 | Positive | 1 | 1 | 1 |

The percentage of neoplastic cells with cytoplasmic/membrane NY-ESO-1 reactivity was registered. The intensity of positive staining was scored as 1 if neatly visible at magnification 20-40X, 2 if neatly visible at magnification 10X, and 3 if neatly visible at magnification 4X (ocular 10X). Tumor was considered positive if at least 1% of neoplastic cells were reactive. The ‘NY-ESO-1 score’ was calculated as the percentage of positive cells multiplied for the intensity score.
