# Supplementary figures and images for: Immune landscape and in vivo immunogenicity of NY-ESO-1 tumor antigen in advanced neuroblastoma patients
Source: BMC Cancer. 2018 Oct 16;18:983. doi: 10.1186/s12885-018-4910-8 (PMC6192300; doi:10.1186/s12885-018-4910-8)

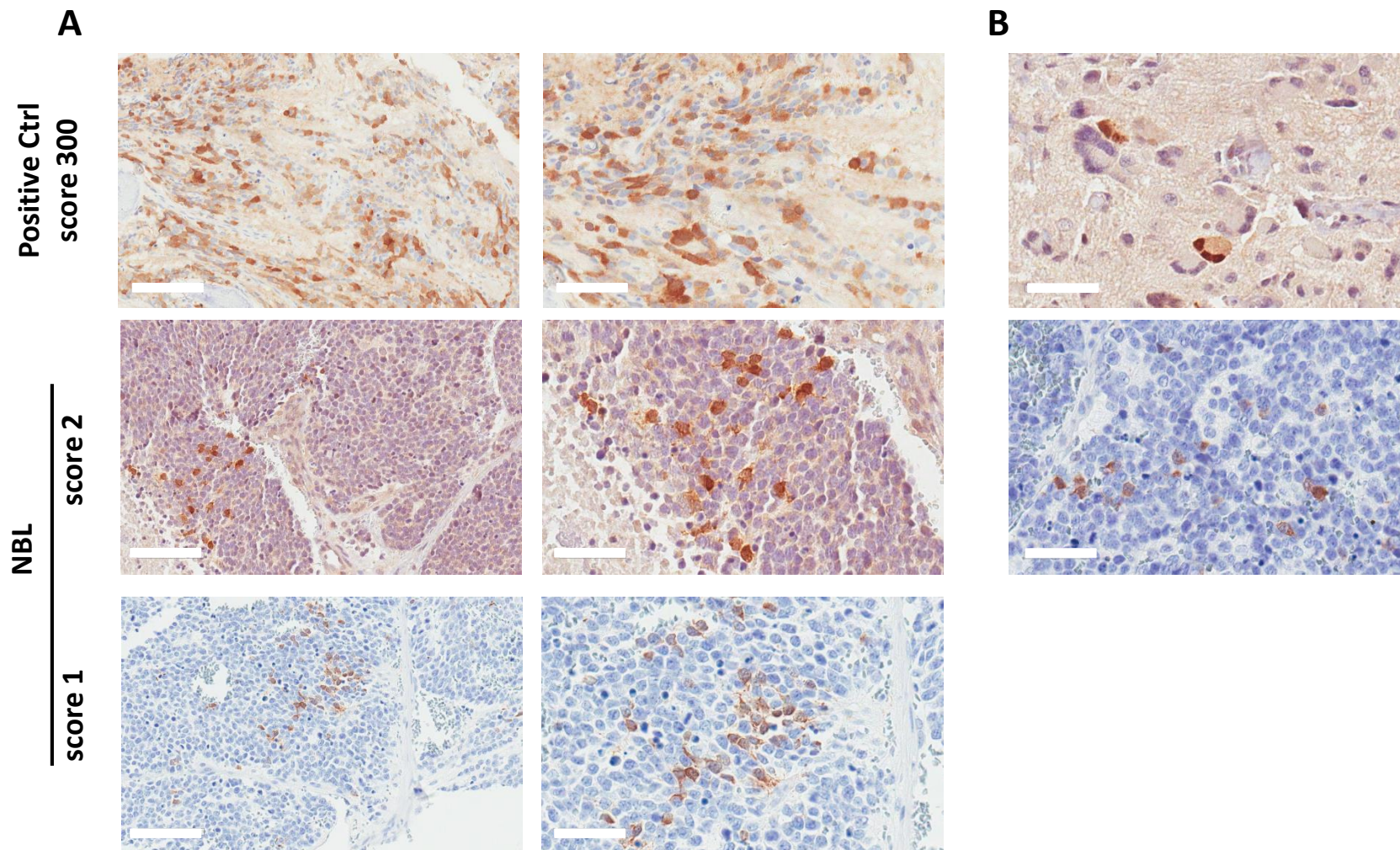

Figure S1

Supplement: Supplementary file 2 — Figure S1. NY-ESO-1 expression in NBL. IHC was performed on FFPE tumor samples obtained before the time of study entry. (A) Representative expression of the NY-ESO-1 marker in a primary cutaneous melanoma (Me5810, positive control) and in two NBL tumors scored as 1 and 2 (for details see Methods and Additional file 1 Table 1S). Scale bar = 200 μm, left panels; scale bar = 50 μm, right panels. (B) NY-ESO-1 expression in NBL tumor cells is detectable both in differentiating and undifferentiating NBL tumors (upper and lower panel respectively). Scale bar = 50 μm. (PDF 512 kb) [file 12885_2018_4910_MOESM2_ESM.pdf]

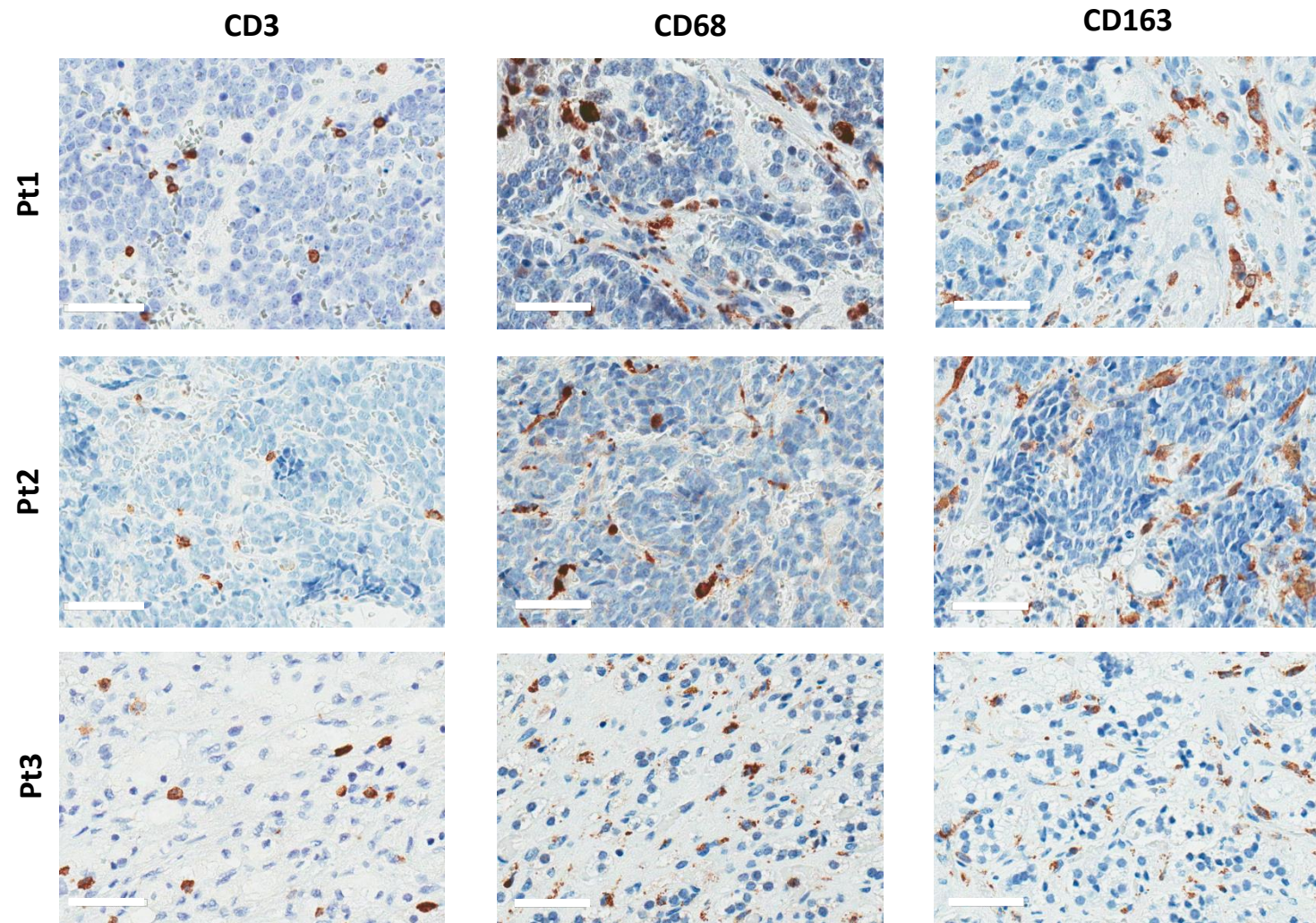

Figure S2

Supplement: Supplementary file 3 — Figure S2. Presence of immune infiltrating cells in NBL before the vaccination. IHC was performed on consecutive sections of FFPE tumor samples. Representative IHC images of intratumoral CD3, CD68 and CD163 positive cells of the tumors of the three enrolled patients: Pt1, 2, 3. Scale bar = 50 μm. (PDF 527 kb) [file 12885_2018_4910_MOESM3_ESM.pdf]

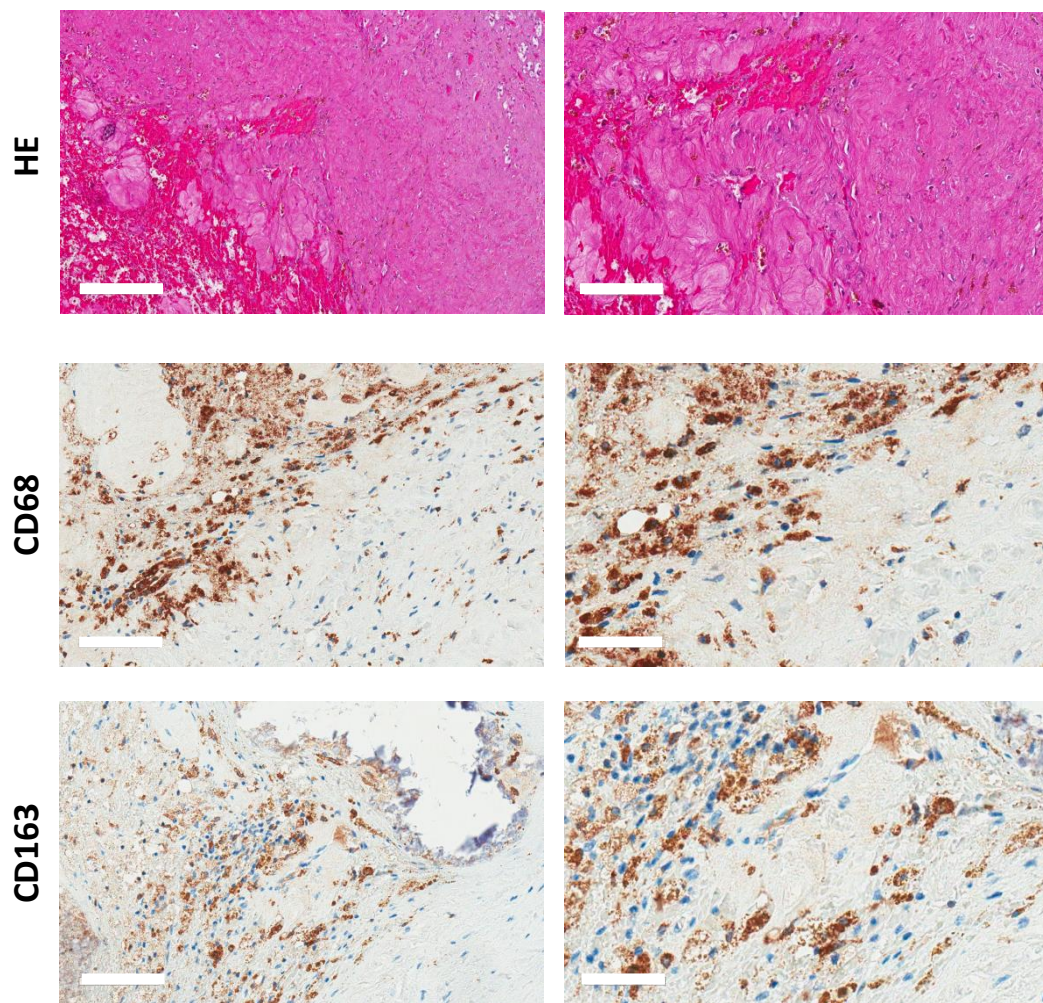

Figure S3

Supplement: Supplementary file 4 — Figure S3. NBL tumor of Patient #3 showed area with pathological response to chemotherapy. HE and IHC analysis of the CD68 and CD163 macrophage markers of pre-vaccine, post-chemotherapy FFPE tumor of Patient #3. Images representative for tumor area displaying post-treatment changes with sclerohyalinosis, fibrous reaction with macrophages, and hemosiderin are reported. For HE scale bar = 200 μm, left panel, and 100 μm, right panel; for the IHC of CD68 and CD163, scale bar = 100 μm, left panel, and 50 μm right panel. (PDF 470 kb) [file 12885_2018_4910_MOESM4_ESM.pdf]

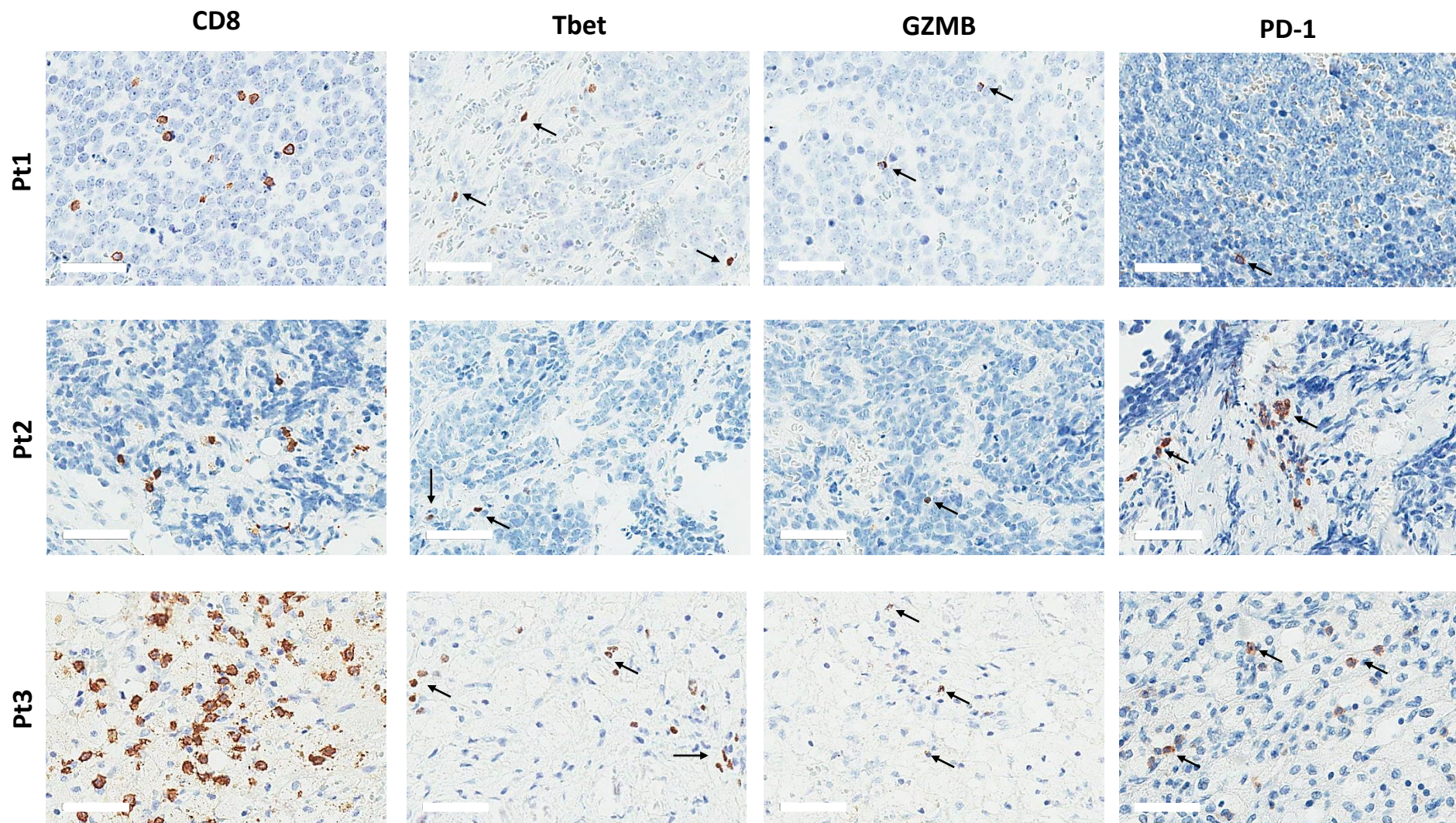

Figure S4

Supplement: Supplementary file 5 — Figure S4. Characterization of CD8 infiltrating cells in NBL before the vaccination. IHC was performed on consecutive sections of FFPE tumor samples that were stained for Tbet, GZMB and PD-1 markers. Examples of immune infiltrating cells with nuclear Tbet, granular cytoplasmic GZMB staining or membrane PD-1 staging of are indicated by the arrows. Scale bar = 50 μm. (PDF 717 kb) [file 12885_2018_4910_MOESM5_ESM.pdf]
